# Supplementary material for: Record high-Tc and large practical utilization level of electric polarization in metal-free molecular antiferroelectric solid solutions
Source: Nat Commun. 2022 Sep 10;13:5329. doi: 10.1038/s41467-022-33039-9 (PMC9464199; doi:10.1038/s41467-022-33039-9)
Supplement: Supplementary file 1 — Supplementary Information [file 41467_2022_33039_MOESM1_ESM.pdf]

Record High-T_c and Large Practical Utilization Level of Electric Polarization in Metal-Free Molecular Antiferroelectric Solid Solutions

Haojie Xu, Wuqian Guo, Yu Ma, Yi Liu, Xinxin Hu, Lina Hua, Shiguo Han, Xitao Liu, Junhua Luo,* and Zhihua Sun*

Experimental Procedures

Synthesis of CMX (X=Cl, Br, I)

All the chemical reagents and solvents were purchased and used without further purification. Compound CMX (CM is cyclohexylmethylammonium, X = Cl, Br I) was synthesized from the concentrated aqueous HX solution containing an appropriate amount of cyclohexanemethylamine. Firstly, cyclohexanemethylamine (~1.5 ml) was dissolved in HX solution (20ml) by heating under constant magnetic stirring to a colorless solution, which yielded the colorless powder precipitation after cooling down to room temperature. Subsequently, the saturated solutions of CMX were prepared after the continuous stirring for 60 min at ~353 K, and the colorless sheet crystals were obtained by the temperature-cooling method with a cooling rate of ~1 K/day.

Synthesis of $CMBr_xCl_{1-x}$ and $CMBr_xI_{1-x}$ ($0 < x < 1$)

The series of binary $CMBr_xI_{1-x}$ and $CMBr_xCl_{1-x}$ ($0 < x < 1$) solid solutions were prepared by combining stoichiometric CMB ($x \times 5$ mmol) and CMC/CMI ($(1-x) \times 5$ mmol) into gamma-butyrolactone/water (10 ml) to get a clear solution. After slow cooling at a rate of 1 K/day, the colorless sheet crystals can be obtained (Supplementary Fig. 1). In this work, we synthesized the samples with $CMBr_xI_{1-x}$ of x = 0, 0.5, 1 and $CMBr_xCl_{1-x}$ of x = 0, 0.2, 0.4, 0.6, 0.7, 0.8, 0.9, 1.

Elemental analysis measurements

The X-ray photoelectron spectroscopy (XPS) was performed on ESCALAB 250Xi (ThermoFisher) (Supplementary Fig. 2). Crystal samples with different components are radiated using X-rays, and the elements are quantitatively analyzed by excitation of photoelectrons. The experimental mass fractions of Cl/Br/I for $CMBr_xCl_{1-x}$ and $CMBr_xI_{1-x}$ match well with the theoretical ones.

Powder X-ray diffraction

Powder X-ray diffraction (PXRD) data were measured on a Rigaku Mini Flex 600 Powder X-ray diffractometer at room temperature that can confirm the phase purity of the crystal, and the diffraction patterns were collected in the 2θ range of 5°-40° with a step size of 0.02° (Supplementary Fig. 3).

Single crystal structure determination

Single crystal X-ray diffraction data of $CMBr_xCl_{1-x}$ and $CMBr_xI_{1-x}$ were collected on a D8 and Agilent Technologies SuperNova Dual Wavelength CCD diffractometer using the Mo Kα radiation at different temperatures. The direct method solved all the crystal structures and then refined them by the full-matrix least-squares refinements on F^2 using *SHELXLTL* 97 software package. The non-hydrogen atoms were refined anisotropically based on all reflections with $I > 2\sigma(I)$, and the hydrogen atoms were generated by geometrical considerations and placed at their idealized positions. Crystallographic data and structure refinements for $CMBr_xCl_{1-x}$ and $CMBr_xI_{1-x}$ are given in Supplementary Table 1, and CCDC 2167457-2167467 contain the crystallographic data in this paper, which can be obtained free of charge from https://www.ccdc.cam.ac.uk/.

Thermal analysis

Differential scanning calorimetry (DSC) measurement was performed on the NETZSCH DSC 200 F3 in the temperature range of 273−500 K. The crystalline samples were placed in aluminum crucibles that were heated and cooled with a rate of 20 K/min under the nitrogen atmosphere (Supplementary Fig. 6). Thermogravimetric analysis (TGA) was performed on STA449C Thermal Analyser ranging from room temperature to 900 ºC with a heating rate of 15 ºC/min. The thermogravimetric measurement reveals that CMC possesses high thermal stability up to ∼530 K, and the differential thermal analysis curve indicates that CMC has a phase transition at 453 K. What greatly favors its potential application as the switchable molecular dielectric is that its melting temperature is far beyond T_c.

Optical axes and ferroelastic domains measurements

The conoscopic images and ferroelastic domains were measured on Nikon Eclipse LV 100N POL. The electric polar crystallographic axis direction of the crystal can be judged by a polarized light microscope. In detail, we determined the optical axial plane by measuring the dual optical axes of biaxial crystal under the conoscope. Based on the electric polarization direction of the orthorhombic system perpendicu-

lar to the optical axial plane, we preliminarily determined the polarization direction and prepared the electrodes. In addition, ferroelastic domains of this binary solid-solution family of $CMBr_xI_{1-x}$ and $CMBr_xCl_{1-x}$have been observed and the results are presented in Supplementary Figure 9.

Dielectric and ferroelectric measurements

During the dielectric and ferroelectric measurements, thin single-crystal plate samples with the thickness of ~0.8 mm were prepared, and silver conductive paste deposited on the plate surfaces were used as top and bottom electrodes. The dielectric constants (ε') and dielectric loss were measured using the two-probe AC impedance method with an Impedance Analyzer (TH2828A) over the frequency range from 500 Hz to 1 MHz. Moreover, the variable temperature current density *versus* electric field (*J-E*) curves and the polarization *versus* electric field (*P-E*) hysteresis loops were recorded with a ferroelectric analyzer (Radiant Precision Premier Ⅱ).

Second harmonic generation (SHG) measurement:

The crystal samples of $CMBr_xCl_{1-x}$ and $CMBr_xI_{1-x}$ with the polished surface were used to measure the SHG properties. The SHG properties were investigated on single-crystal samples with a homemade Q-switched Nd:YVO_4 laser at 1064 nm (full-width at half maximum of 100 fs, repetition rate of 1 kHz). The laser beam is linearly polarized and focuses on the sample with the radius of ~220 μm. The crystal sample with dimension of ~2×2×0.5 mm^3 was used to measure SHG properties. No SHG signal was measured at room temperature, suggesting that the solid solution compounds crystallize in the centro-symmetry space group.

Supplementary Figures

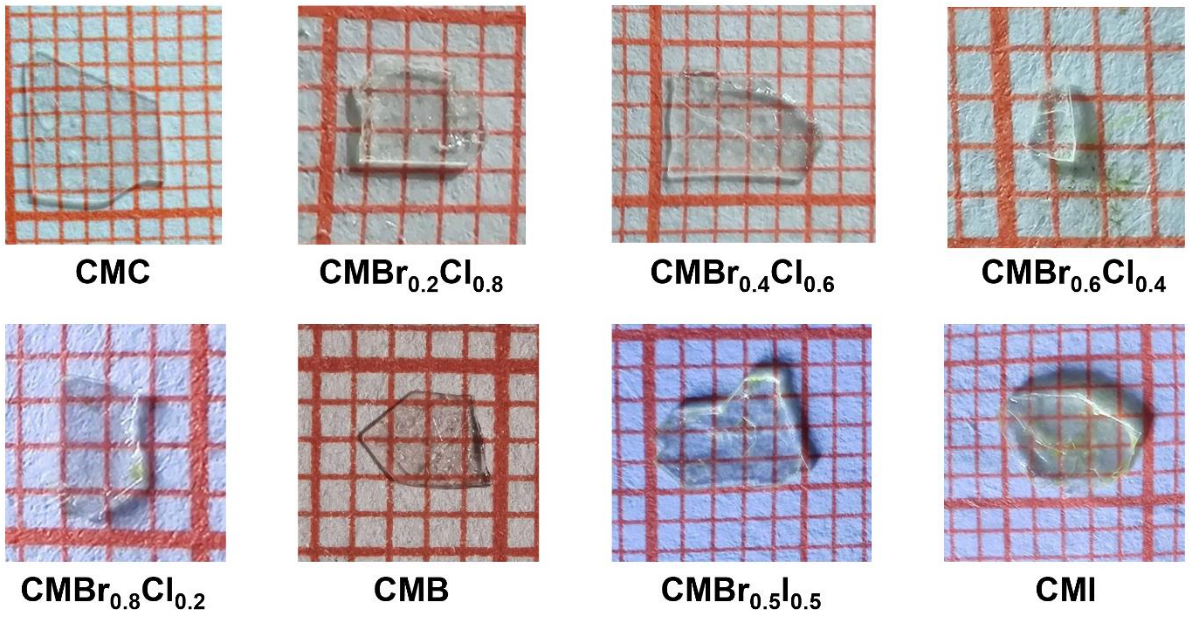

Supplementary Fig. 1 | Colorless bulk crystals of $CMBr_xCl_{1-x}$ and $CMBr_xI_{1-x}$ for the selected compositions.

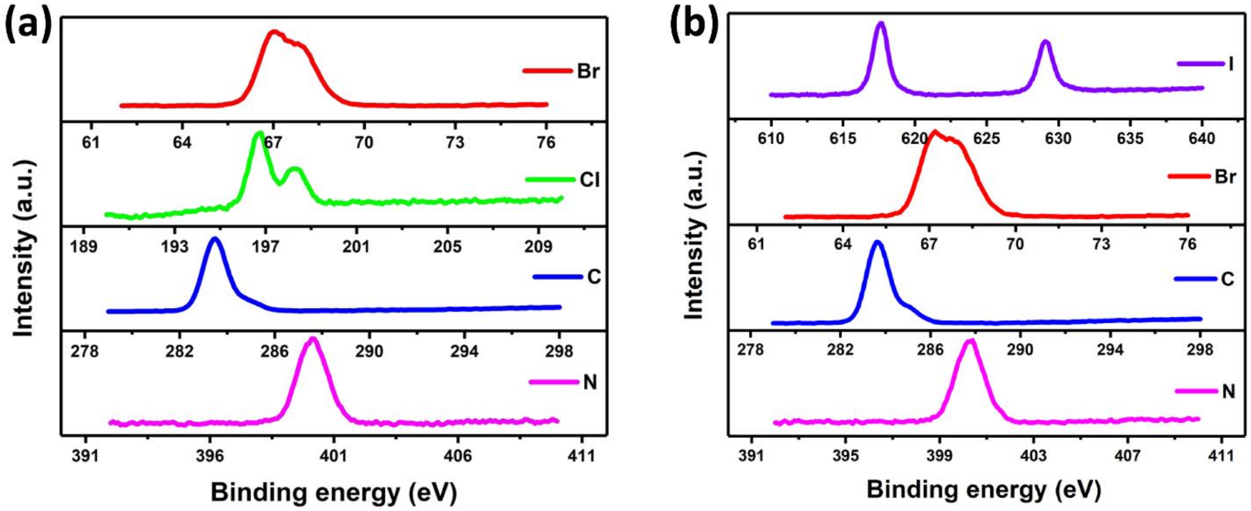

Supplementary Fig. 2 | The X-ray photoelectron spectroscopy (XPS) of (a) $CMBr_xCl_{1-x}$ and (b) $CMBr_xI_{1-x}$ for selected compositions. The proportion of Cl/Br/I can be obtained by calculating the ratio of the peak area to the sensitivity factor.

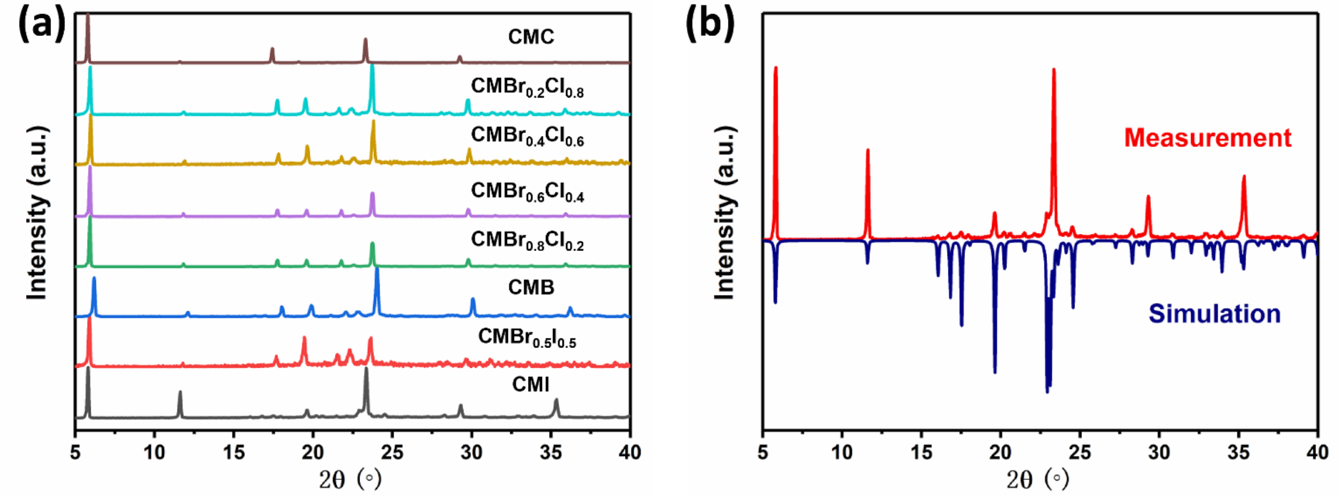

Supplementary Fig. 3 | (a) Powder X-ray diffraction (PXRD) patterns of $CMBr_xCl_{1-x}$ and $CMBr_xI_{1-x}$ ($0 \le x \le 1$). (b) Simulated and experimental PXRD patterns of CMC measured at room temperature, verifying the phase purity of the bulk powders.

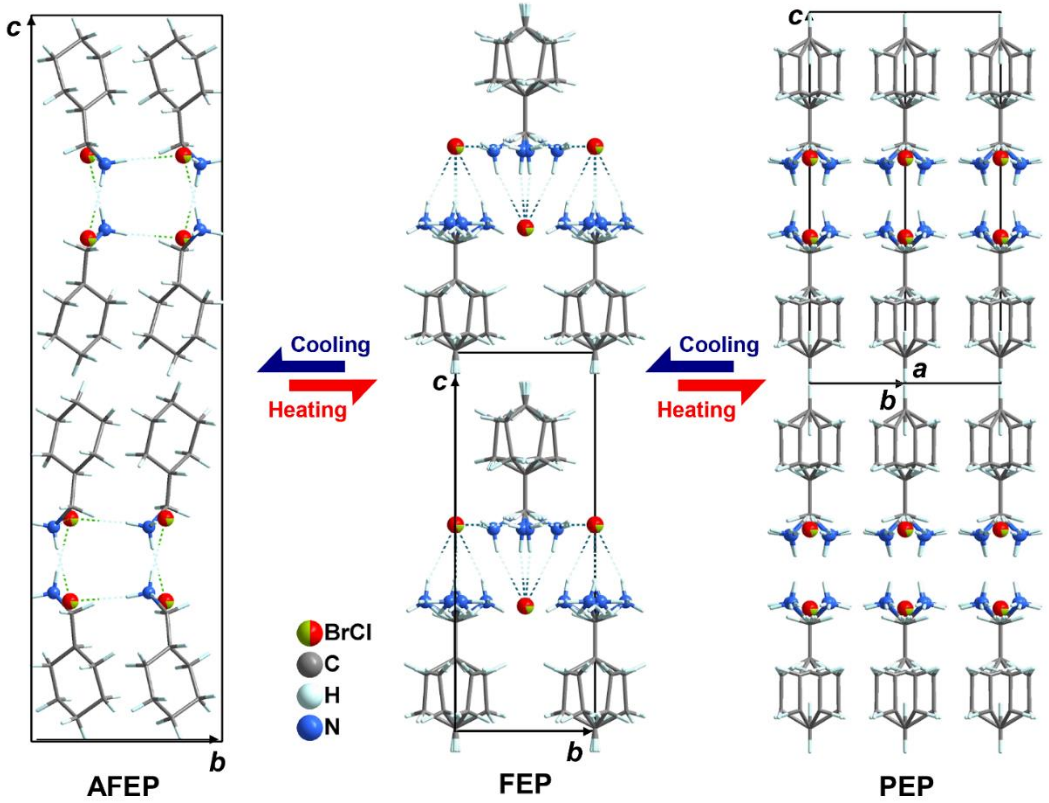

Supplementary Fig. 4 | Variable temperature crystal structures of $CMBr_{0.8}Cl_{0.2}$, which is a typical example of the AFEP-FEP-PEP phase transition in $CMBr_xCl_{1-x}$ and $CMBr_xI_{1-x}$ solid solutions.

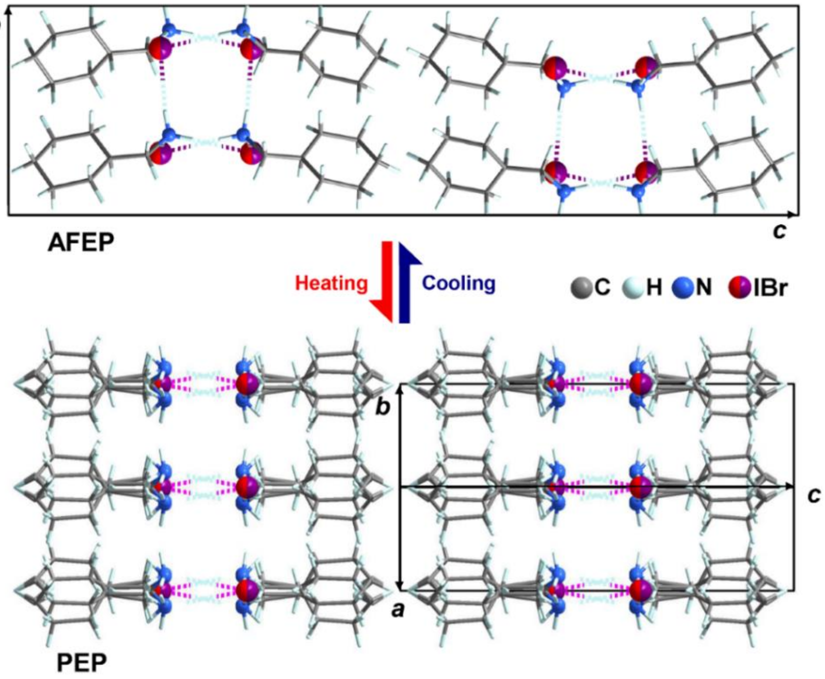

Supplementary Fig. 5 | Variable temperature crystal structures of $CMBr_{0.5}I_{0.5}$, which is a typical example of the AFEP-PEP phase transition in $CMBr_xCl_{1-x}$ and $CMBr_xI_{1-x}$ solid solutions.

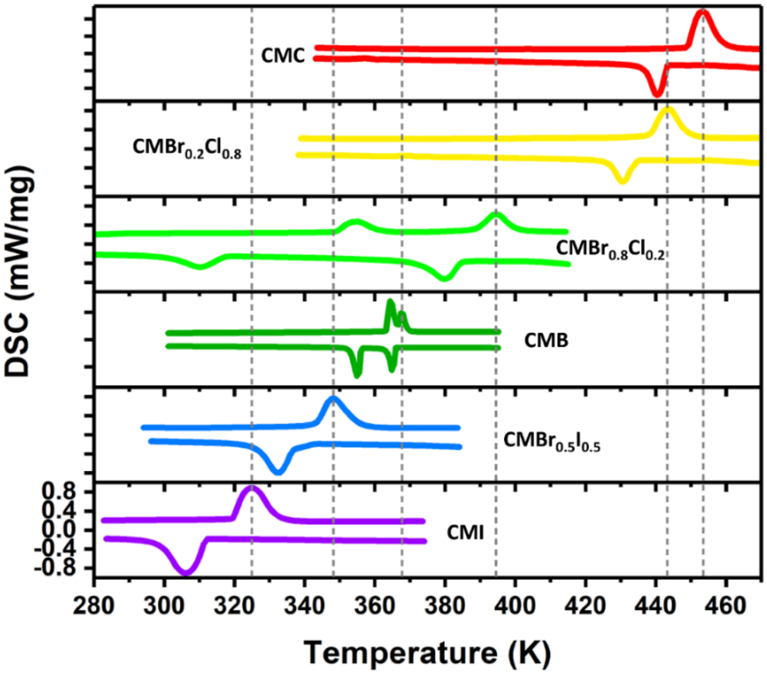

Supplementary Fig. 6 | DSC curves with the heating/cooling rate of 20 K/min for the selected compositions.

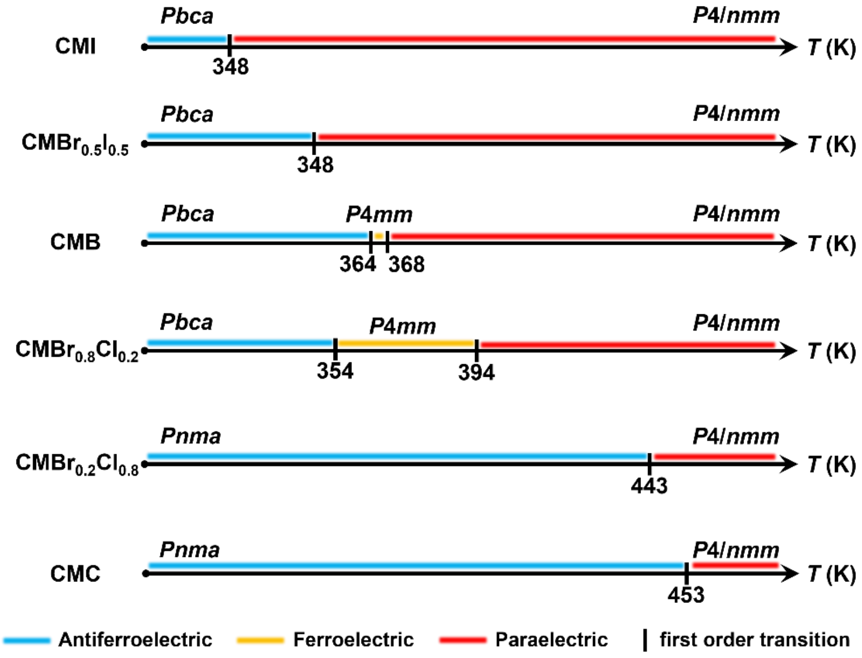

Supplementary Fig. 7 | Phase transition properties and space groups of $CMBr_xI_{1-x}$ and $CMBr_xCl_{1-x}$ solid solutions.

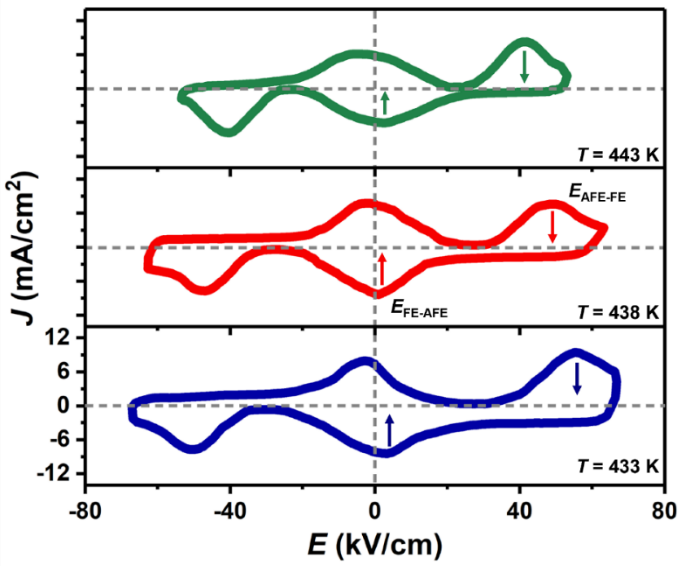

Supplementary Fig. 8 | *J*−*E* curves measured at different temperatures. The coercive electric fields to switch dipolar moments, including the forward switching field from AFEP-to-FEP transition ($E_{\text{AFE-FE}}$) and backward switching field from FEP-to-AFEP transition ($E_{\text{FE-AFE}}$), are estimated as ~2 and ~49 kV/cm at 438 K, respectively. It is notable that both $E_{\text{AFE-FE}}$ and $E_{\text{FE-AFE}}$ show temperature-dependent behavior.

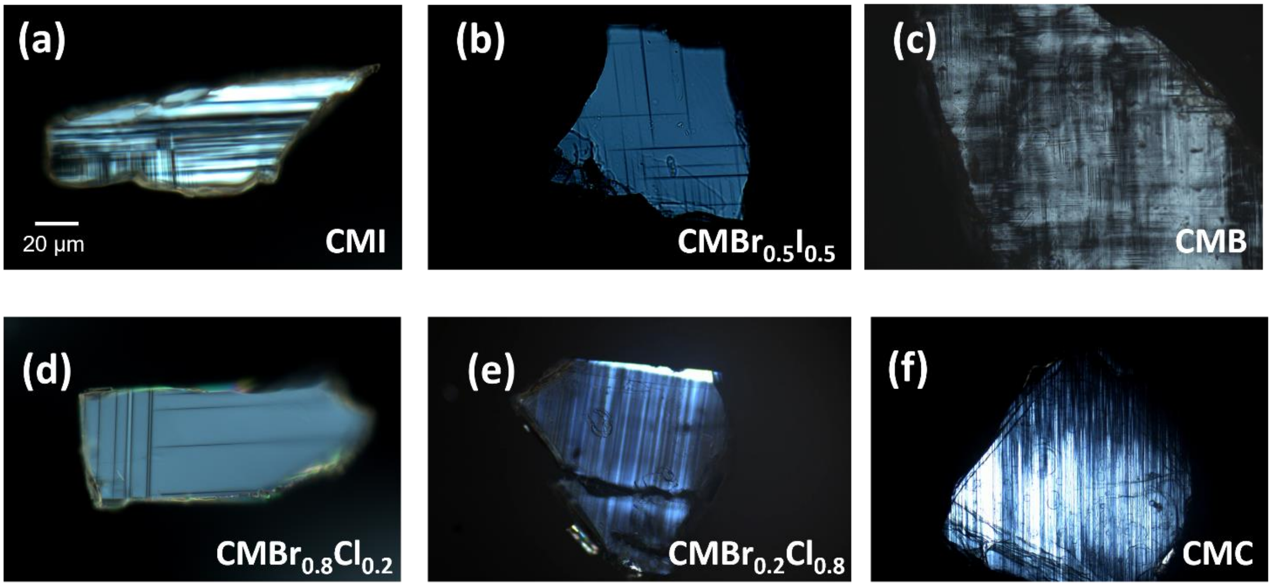

Supplementary Fig. 9 | Ferroelastic domains observed in the binary solid-solution family. Ferroelastic domain structure of (a) CMI, (b) $CMBr_{0.5}I_{0.5}$, (c) CMB, (d) $CMBr_{0.8}Cl_{0.2}$, (e) $CMBr_{0.2}Cl_{0.8}$ and (f) CMC at room temperature.

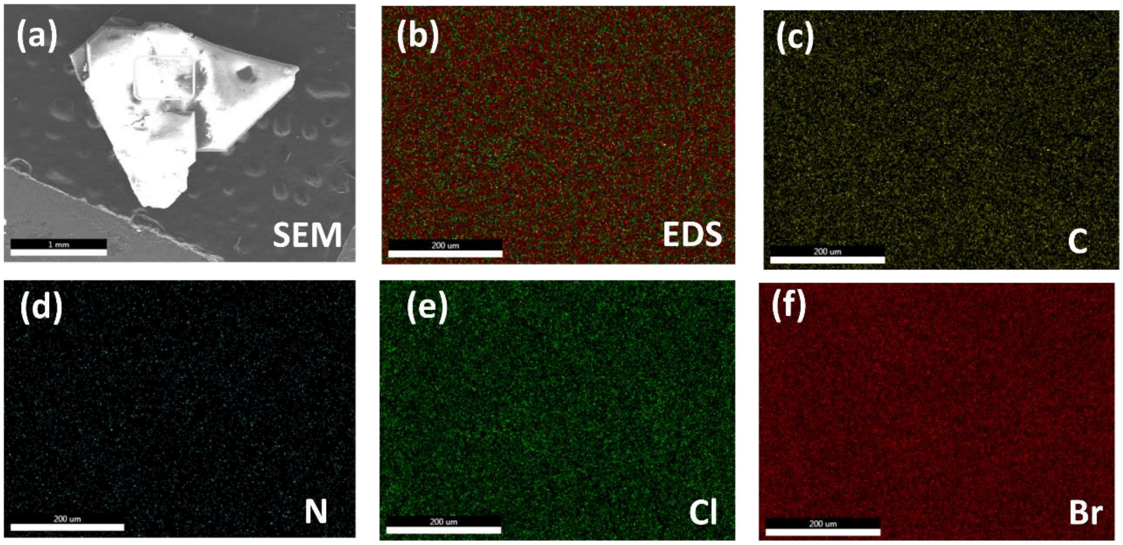

Supplementary Fig.10 | Homogeneity of the proportion of the binary solid-solution $CMBr_{0.8}Cl_{0.2}$. (a) FESEM images of $CMBr_{0.8}Cl_{0.2}$ single crystal. (b) EDS elemental mapping of $CMBr_{0.8}Cl_{0.2}$ single crystal. (c-f) The distribution of C, N, Cl, and Br in the whole selected area reveals that the elements are uniformly dispersed on the single crystals.

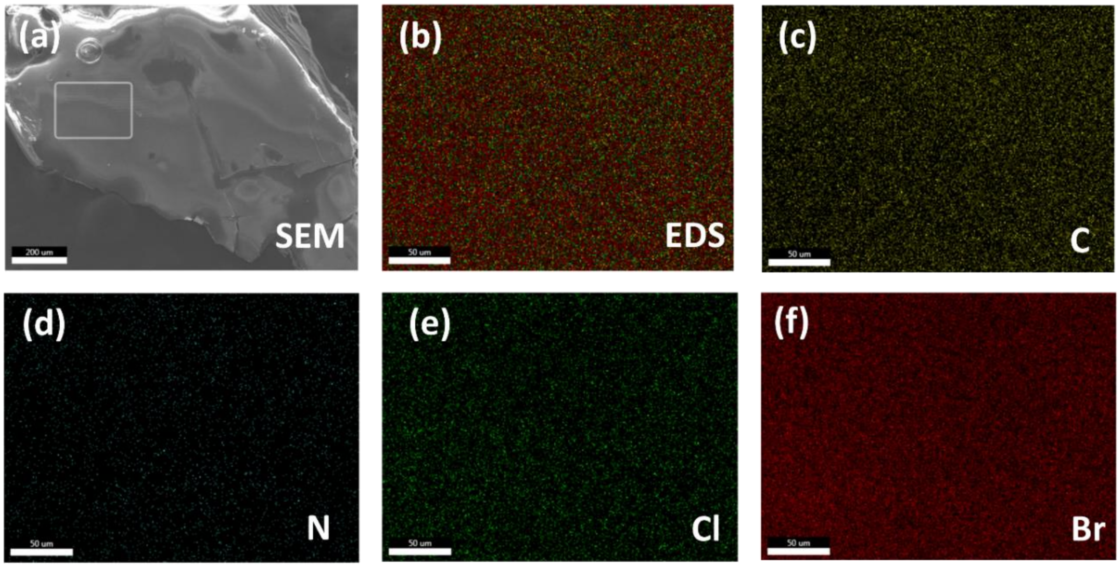

Supplementary Fig. 11 | Homogeneity of the proportion of the binary solid-solution $CMBr_{0.2}Cl_{0.8}$. (a) FESEM images of $CMBr_{0.2}Cl_{0.8}$ single crystal. (b) EDS elemental mapping of $CMBr_{0.2}Cl_{0.8}$ single crystal. (c-f) The distribution of C, N, Cl, and Br in the whole selected area reveals that the elements are uniformly dispersed on the single crystals.

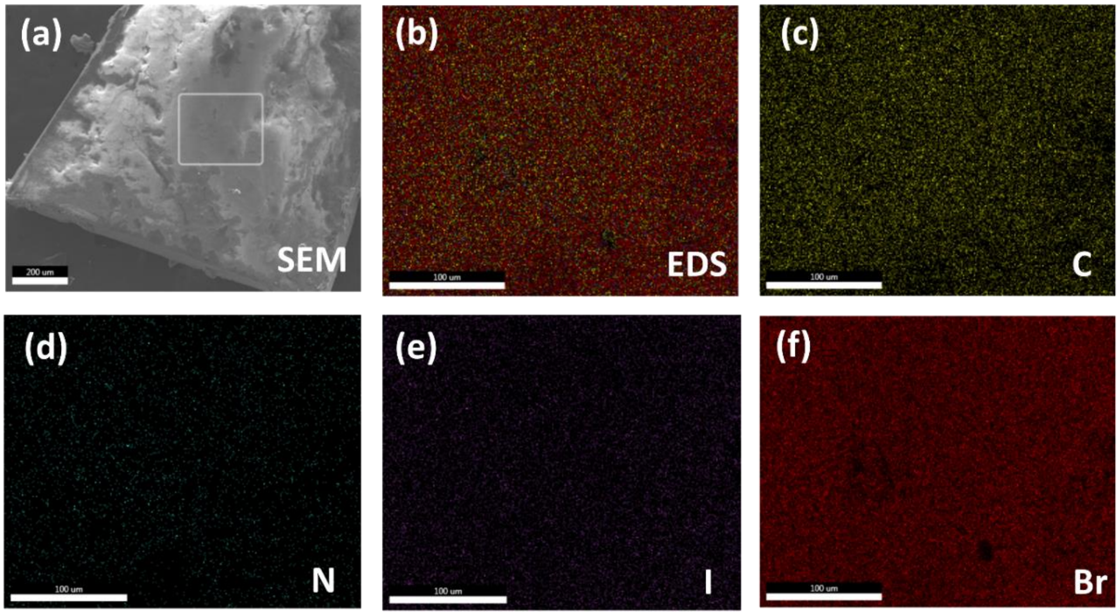

Supplementary Fig. 12 | Homogeneity of the proportion of the binary solid-solution $CMBr_{0.5}I_{0.5}$. (a) FESEM images of $CMBr_{0.5}I_{0.5}$ single crystal. (b) EDS elemental mapping of $CMBr_{0.5}I_{0.5}$ single crystal. (c-f) The distribution of C, N, I, and Br in the whole selected area reveals that the elements are uniformly dispersed on the single crystals.

**Supplementary Table 1 | ** Crystal data and structure refinement of $CMBr_xCl_{1-x}$ and $CMBr_xI_{1-x}$ for selected compositions at room temperature.

Composition	CMI	$CMBr_{0.5}I_{0.5}$	CMB	$CMBr_{0.8}Cl_{0.2}$	$CMBr_{0.4}Cl_{0.6}$	$CMBr_{0.2}Cl_{0.8}$	CMC
Empirical formula	$C_7H_{16}IN$	$C_7H_{16}Br_{0.5}I_{0.5}N$	$C_7H_{16}BrN$	$C_7H_{16}Br_{0.8}Cl_{0.2}N$	$C_7H_{16}Br_{0.4}Cl_{0.6}N$	$C_7H_{16}Br_{0.8}Cl_{0.2}N$	$C_7H_{16}ClN$
Temperature (*K*)	300.02	299.84	298	300.03	299.03	295.66	297.99
Crystal system	orthorhombic	orthorhombic	orthorhombic	orthorhombic	orthorhombic	orthorhombic	orthorhombic
Space group	*Pbca*	*Pbca*	*Pbca*	*Pbca*	*Pnma*	*Pnma*	*Pnma*
a (Å)	8.5127(5)	8.1913(4)	8.1818(2)	8.1334(5)	30.695(5)	30.578(5)	30.473(4)
b (Å)	8.0799(4)	7.9484(4)	7.9363(3)	7.8809(7)	5.3756(8)	5.3565(9)	5.3580(8)
c (Å)	30.5267(16)	30.0141(12)	30.0031(10)	29.982(3)	5.7937(9)	5.6534(10)	5.6102(6)
Volume ($Å^3$)	2099.7(2)	1954.15(16)	1948.20(11)	1921.8(3)	956.0(3)	926.0(3)	916.0(2)
Z	8	8	8	8	4	4	4
$\rho_{cal.}$ (g/cm^3)	1.525	1.479	1.324	1.280	1.163	1.137	1.085
F(000)	944.0	872 .0	800.0	771.0	357.0	342.0	328.0
Index ranges	$-11 \le h \le 10$, $-10 \le k \le 10$, $-38 \le l \le 39$	$-10 \le h \le 9$, $-10 \le k \le 10$, $-38 \le l \le 37$	$-10 \le h \le 10$, $-10 \le k \le 10$, $-38 \le l \le 38$	$-10 \le h \le 10$, $-10 \le k \le 9$, $-38 \le l \le 38$	$-39 \le h \le 39$, $-6 \le k \le 6$, $-7 \le l \le 7$	$-35 \le h \le 36$, $-6 \le k \le 6$, $-6 \le l \le 6$	$-38 \le h \le 39$, $-6 \le k \le 6$, $-7 \le l \le 7$
Reflections collected	14412	13799	13666	12340	13180	5282	6292
Independent reflections	2411 [R_{int} = 0.2084]	2247 [R_{int} = 0.1287]	2243 [R_{int} = 0.0984]	2215 [R_{int} = 0.0812]	1215 [R_{int} = 0.1723]	915 [R_{int} = 0.1450]	1181 [R_{int} = 0.1164]
Data/restraints/parameters	2411/0/84	2247/0/78	2243/0/89	2215/104/83	1215/58/71	915/18/71	1181/15/71
GOF	1.042	1.010	1.024	1.041	0.938	1.026	1.036
Final *R* indexes [I>=2σ (I)]	R_1 = 0.0949 wR_2 = 0.2269	R_1 = 0.0666 wR_2 = 0.1651	R_1 = 0.0528 wR_2 = 0.1107	R_1 = 0.0671 wR_2 = 0.1884	R_1 = 0.0664 wR_2 = 0.1616	R_1 = 0.0727 wR_2 = 0.1519	R_1 = 0.0668 wR_2 = 0.1849
Final *R* indexes [all data]	R_1 = 0.1955 wR_2 = 0.2994	R_1 = 0.1550 wR_2 = 0.2151	R_1 = 0.1252 wR_2 = 0.1428	R_1 = 0.1137 wR_2 = 0.2249	R_1 = 0.1648 wR_2 = 0.2258	R_1 = 0.1607 wR_2 = 0.1959	R_1 = 0.1154 wR_2 = 0.2223

Supplementary Table 2 | Lattice parameter of $CMBr_xCl_{1-x}$ and $CMBr_xI_{1-x}$ for selected compositions in low-temperature phase and high-temperature phase.

Composition	CMI		$CMBr_{0.5}I_{0.5}$		CMB		
T (K)	300.02	339.88	299.84	401.45	298	367	380
Crystal system	orthorhombic	tetragonal	orthorhombic	tetragonal	orthorhombic	tetragonal	tetragonal
Space group	*Pbca*	*P*4/*nmm*	*Pbca*	*P*4/*nmm*	*Pbca*	*P*4*mm*	*P*4/*nmm*
a (Å)	8.5127(5)	5.8466(6)	8.1913(4)	5.7847(7)	8.1818(2)	5.6871(5)	5.6978(5)
b (Å)	8.0799(4)	5.8466(6)	7.9484(4)	5.7847(7)	7.9363(3)	5.6871(5)	5.6978(5)
c (Å)	30.5267(16)	15.6494(14)	30.0141(12)	15.601(2)	30.0031(10)	15.4564(17)	15.454(3)
α (degree)	90	90	90	90	90	90	90
β (degree)	90	90	90	90	90	90	90
γ (degree)	90	90	90	90	90	90	90
V (Å^3)	2099.7(2)	534.94(12)	1954.15(16)	522.06(14)	1948.20(11)	499.91(10)	501.72(12)
Composition	$CMBr_{0.8}Cl_{0.2}$			CMC			
T (K)	300.03	360.12	400.03	297.99	446.85		
Crystal system	orthorhombic	tetragonal	tetragonal	orthorhombic	tetragonal		
Space group	*Pbca*	*P*4*mm*	*P*4/*nmm*	*Pnma*	*P*4/*nmm*		
a (Å)	8.1334(5)	5.586(8)	5.652(2)	30.473(4)	5.606(2)		
b (Å)	7.8809(7)	5.586(8)	5.652(2)	5.3580(8)	5.606(2)		
c (Å)	29.982(3)	15.16(2)	15.545(6)	5.6102(6)	15.421(6)		
α (degree)	90	90	90	90	90		
β (degree)	90	90	90	90	90		
γ (degree)	90	90	90	90	90		
V (Å^3)	1921.8(3)	473.0(15)	496.5(4)	916.0(2)	484.6(4)		

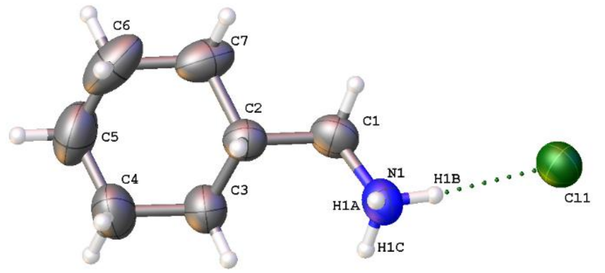

Atom number for crystal **CMC** at AFEP (298 K)

Supplementary Table 3 | Bond lengths for crystal **CMC** at AFEP (298 K).

Atom	Atom	Length/Å	Atom	Atom	Length/Å
N1	C1	1.476(5)	C6	C7	1.534(9)
C2	C1	1.518(5)	C6	C5	1.493(16)
C2	C7	1.533(6)	C3	C4	1.530(7)
C2	C3	1.501(7)	C5	C4	1.493(10)

Supplementary Table 4 | Bond angles for crystal **CMC** at AFEP (298 K).

Atom	Atom	Atom	Angle/°	Atom	Atom	Atom	Angle/°
C1	C2	C7	109.3(4)	C6	C7	C2	110.7(5)
C3	C2	C1	110.8(3)	C2	C3	C4	112.8(6)
C3	C2	C7	109.7(4)	C4	C5	C6	110.5(8)
N1	C1	C2	114.0(3)	C5	C4	C3	111.4(7)
C5	C6	C7	112.3(10)				

Supplementary Table 5 | N-H···Cl for crystal **CMC** at AFEP (298 K).

D-H···A	d(D-H)	d(H···A)	<DHA	d(D···A)
N1-H1A···Cl1[#1]	0.890	2.327	163.47	3.191
N1-H1B···Cl1	0.890	2.266	170.66	3.148
N1-H1C···Cl1[#2]	0.890	2.315	168.04	3.191

[#1]-*x*+1/2, *y*-1/2, *z*+1/2; [#2]-*x*+1/2, *y*+1/2, *z*+1/2.

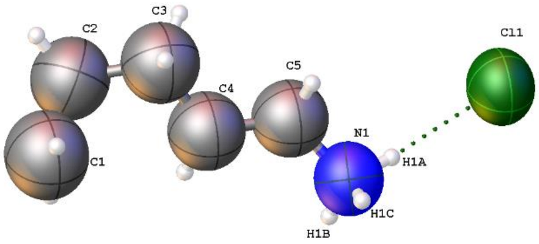

Atom number for crystal **CMC** at PEP (447 K)

Supplementary Table 6 | Bond lengths for crystal **CMC** at PEP (447 K).

Atom	Atom	Length/Å	Atom	Atom	Length/Å
C5	C4	1.475(7)	C2	C1	1.498(8)
C5	N1	1.437(8)	C4	C3	1.485(7)
C2	C3	1.602(9)			

Supplementary Table 7 | Bond angles for crystal **CMC** at PEP (447 K).

Atom	Atom	Atom	Angle/°	Atom	Atom	Atom	Angle/°
N1	C5	C4	126.6(4)	C5	C4	C3	118.9(3)
C1	C2	C3	106.1(6)	C4	C3	C2	105.9(5)

Supplementary Table 8 | N-H···Cl for crystal **CMC** at PEP (447 K).

D-H···A	d(D-H)	d(H···A)	<DHA	d(D···A)
N1-H1A···Cl1	0.890	2.224	164.06	3.089
N1-H2B···Cl1[#1]	0.890	2.368	159.01	3.214
N1-H2C···Cl1[#2]	0.890	2.418	178.39	3.308

[#1] -*x*+1, -*y*+1, -*z*+2; [#2] -*x*, -*y*+1, -*z*+2.

Supplementary Table 9 | Phase transition temperatures and symmetry changes of some known molecular antiferroelectric materials.

Compound	Symmetry change (#)	Phase transition temperatures/K	Physical properties	Ref.
CMC	*Pnma* (AFE)→ *P*4/*nmm* (PE)	453	*P-E* hysteresis loop	This
CMI	*Pbca* (AFE) →*P*4/*nmm* (PE)	324	Dielectric	work
CMB	*Pbca* (AFE)→*P*4*mm* (FE) →*P*4/*nmm* (PE)	364, 368	*P-E* hysteresis loop	1
$NH_4H_2PO_4$	$P2_12_12_1$ (AFE) →*I*42*d* (PE)	147	Dielectric	2-3
$Cu(HCO_2)_2·4H_2O$	$P2_1/c$ (AFE)→?	235	*P-E* hysteresis loop	4
$(CH_3)_3NCH_2COO·H_3PO_4$	$P2_1/c$ (AFE)→$P2_1/m$ (PE)	365	Dielectric	5
MeHPLN	$P2_1/c$ (AFE)→*C*2/*c* (PE)	42	X-ray and neutron diffraction	6
[H-55dmbp][Hca] salt	*P*-1(AFE)→*P*-1(PE)	318	*P-E* hysteresis loop	7
$(i\text{-}BA)_2CsPb_2Br_7$	*Pmnb* (AFE)→*Cmca* (PE)	353	*P-E* hysteresis loop	8
$(3\text{-pyrrolinium})CdBr_3$	$Cmc2_1$ (FE)→*C*2/*m* (AFE)→*Cmcm* (PE)	235, 244	*P-E* hysteresis loop	9
$(i\text{-}PA)_2CsPb_2Br_7$	$Cmc2_1$ (FE)→*Pbcm* (AFE)→*I*4/*mmm* (PE)	321, 350	*P-E* hysteresis loop	10
$(BA)_2(EA)_2Pb_3I_{10}$	$Cmc2_1$ (FE)→*Pbca* (AFE)→*I*4/*mmm* (PE)	322, 363	*P-E* hysteresis loop	11
$p\text{-PBSQ}·2H_2O$	? → *P*-1(PE)	28	Dielectric	12
BSQA	? → $P4_2/mnm$(PE)	40	Dielectric	13
CbxHPLN	? → $P2_1/c$(PE)	40	Dielectric	14
DMTTF-CA	*P*-1(AFE)→*P*-1(PE)	65	Dielectric	15
$[(CH3)_2NH_2]Zn(HCOO)_3$	? → *R*-3*c*(PE)	156	Dielectric	16
Hnpd-Hia	*P*-1(AFE)→*P*-1(PE)	262	Dielectric	17
Per-TBPA	*P*-1(AFE)→*P*-1(PE)	268	Dielectric	18
Hnpd-Hba	*P*-1(AFE)→*P*-1(PE)	328	Dielectric	17
Hnpd-Hfa	*P*-1(AFE)→*P*-1(PE)	338	Dielectric	17
Hnpd-Hca	*P*-1(AFE)→*P*-1(PE)	340	Dielectric	17
$(i\text{-}PA)_2(EA)_2Pb_3I_{10}$	$Cmc2_1$(FE)→*Pmcn*(AFE)→*I*4/*mmm*(PE)	313,340	*P-E* hysteresis loop	19
Br2IMes	*P*-1(AFE)→?	357	Dielectric	20
$HPz\text{-}ClO_4$	*C*2/*m*(AFE)→?	358	Dielectric	21
SQA	$P2_1/m$→*I*4/*m*	373	*P-E* hysteresis loop	7
Hdtbbp-Hca	? → *P*-1(PE)	412	Dielectric	22

[#] antiferroelectric (AFE), ferroelectric (FE), paraelectric (PE)

Supplementary Table 10 | Comparison of the energy conservation properties of some different anti-ferroelectric materials.

Material	Form	*E* (kV/cm)	W_{st} (J/cm^3)	W_{re} (J/cm^3)	η (%)	Reference
This work	single crystal	63	0.54	0.28	52	This work
CMB	single crystal	57	0.23	0.1	43	1
[H-55dmbp][Hca] salt	single crystal	173	0.56	0.51	90	7
$(i\text{-}BA)_2CsPb_2Br_7$	single crystal	94	0.38	0.26	63	8
$(BA)_2(EA)_2Pb_3I_{10}$	single crystal	60	0.18	0.15	83	11
$AgNbO_3$	ceramic	184	7	2.8	40	23
$Pb_{0.97}La_{0.02}(Zr_{0.50}Sn_{0.39}Ti_{0.11})O_3$	ceramic	130	3	1.5	50	24
0.95NBT-0.05BH	ceramic	125	2.7	1.3	48.2	25
$Hf_{0.3}Zr_{0.7}O_2$	film	3260	55	28	51	26

Supplementary References

1. Xu, H. et al. A Metal-Free Molecular Antiferroelectric Material Showing High Phase Transition Temperatures and Large Electrocaloric Effects. *J. Am. Chem. Soc.* **143**, 14379-14385 (2021).
2. Nagamiya, T., On the Theory of the Dielectric, Piezoelectric, and Elastic Properties of $NH_4H_2PO_4$. *Prog. Theor. Phys.* **7**, 275-284 (1952).
3. Schmidt, V. H., Review of Order-Disorder Models for KDP-Family Crystals. *Ferroelectrics* **72**, 157-173 (1987).
4. Okada, K., Antiferroelectric Phase Transition in Copper-Formate Tetrahydrate. *Phys. Rev. Lett.* **15**, 252-254 (1965).
5. Schildkamp, W.; Spilker, J., Structural and Antiferroelectric Phase Transitions in Betaine Phosphate, $(CH_3)_3NCH_2COOH_3PO_4$. *Z KRISTALLOGR* **168**, 159-171 (1984).
6. Kiyanagi, R. et al. Phase Transition Scheme of Isolated Hydrogen-bonded Material h-MeHPLN Studied by Neutron and X-ray Diffraction. *J. Phys. Soc. Jpn.* **74**, 613-620 (2005).
7. Horiuchi, S.; Kumai, R. & Ishibashi, S. Strong Polarization Switching with Low-Energy Loss in Hydrogen-Bonded Organic Antiferroelectrics. *Chem. Sci.* **9**, 425-432 (2018).
8. Wu, Z. et al. Discovery of an Above-Room-Temperature Antiferroelectric in Two-Dimensional Hybrid Perovskite. *J. Am. Chem. Soc.* **141**, 3812-3816 (2019).
9. Li, P. F. et al. Unprecedented Ferroelectric-Antiferroelectric-Paraelectric Phase Transitions Discovered in an Organic-Inorganic Hybrid Perovskite. *J. Am. Chem. Soc.* **139**, 8752-8757 (2017).
10. Li, M. et al. Soft Perovskite-Type Antiferroelectric with Giant Electrocaloric Strength near Room Temperature. *J. Am. Chem. Soc.* **142**, 20744-20751 (2020).
11. Han, S. et al. High-Temperature Antiferroelectric of Lead Iodide Hybrid Perovskites. *J. Am. Chem. Soc.* **141**, 12470-12474 (2019).
12. Takasu, A. I., T. Sugawara, & T. Mochida, J., Observation of Quantum Paraelectricity in an Inter-molecular Ionic Hydrogen-Bonded Crystal of a Squaric Acid Derivative. *J. Phys. Chem. B* **108**, 5527-5531 (2004).
13. Takasu, T. S., & T. Mochida, Dielectric Response in Bisquaric Acid Crystal: Possible Generation of Protonic Soliton in a Quasi-One-Dimensional Hydrogen-Bonded System. *J. Phys. Chem. B* **108**, 18495-18499 (2004).
14. Sugawara, K. D., Y. Moritomo, & Y. Tokura, Organic Paraelectrics Resulting from Tatomeirization Coupled with Proton-Transfer. *Solid State Commun.* **83**, 665-668 (1992).
15. S. Horiuchi, Y. O., R. Kumai, & Y. Tokura, Quantum Phase Transition in Organic Charge-Transfer Complexes. *Science* **299**, 229 (2003).

16. Prashant Jain, at al. Order-Disorder Antiferroelectric Phase Transition in a Hybrid Inorganic-Organic Framework with the Perovskite Architecture. *J. Am. Chem. Soc.* **130**, 10450-10451 (2008).
17. Kobayashi, K. et al. Structure-property relationship of supramolecular ferroelectric [H-66dmbp][Hca] accompanied by high polarization, competing structural phases, and polymorphs. *Chem.-Eur. J.* **20**, 17515-17522 (2014).
18. Jun Harada, M. O., Yukihiro Takahashi, & Tamotsu Inabe. A Collective In-plane Molecular Rotator based on Dibromoiodomesitylene π-Stacks. *J. Am. Chem. Soc.* **137**, 4477-4486 (2015).
19. Han, S. et al. Tailoring of a visible-light-absorbing biaxial ferroelectric towards broadband self-driven photodetection. *Nat. Commun.* **12**, 284 (2021).
20. Jun-ichi Ichikawa, Norihisa Hoshino, Takashi Takeda, Tomoyuki Akutagawa, A Collective In-plane Molecular Rotator based on Dibromoiodomesitylene π-Stacks. *J. Am. Chem. Soc.* **137**, 13155-13160 (2015).
21. Andrzej Katrusiak, Marek Szafrański, Disproportionation of Pyrazine in NH^+···N Hydrogen-Bonded Complexes: New Materials of Exceptional Dielectric Response. *J. Am. Chem. Soc.* **128**, 15775-15785 (2006).
22. Bator, G. et al. Hydrogen bonded NHO chains formed by chloranilic acid (CLA) with 4,4'-di-t-butyl-2,2'-bipyridyl (dtBBP) in the solid state. *Chem. Phys.* **392**, 114-121 (2012).
23. Luo, N. et al. Aliovalent A-site engineered $AgNbO_3$ lead-free antiferroelectric ceramics toward superior energy storage density. *J. Mater. Chem. A* **7**, 14118-14128 (2019).
24. Dan, Y. et al. Energy storage characteristics of $(Pb,La)(Zr,Sn,Ti)O_3$ antiferroelectric ceramics with high Sn content. *Appl. Phys. Lett.* **113**, 063902 (2018).
25. Zhang, L. et al. High energy-storage density under low electric fields and improved optical transparency in novel sodium bismuth titanate-based lead-free ceramics. *J. Eur. Ceram. Soc.* **40**, 71-77 (2020).
26. Park, M. H. et al. Thin $Hf_xZr_{1-x}O_2$ Films: A New Lead-Free System for Electrostatic Supercapacitors with Large Energy Storage Density and Robust Thermal Stability. *Adv. Energy Mater.* **4**, 1400610 (2014).
